# Workplace violence against physicians and nurses in Palestinian public hospitals: a cross-sectional study

**DOI:** 10.1186/1472-6963-12-469

**Published:** 2012-12-20

**Authors:** Mohamad Kitaneh, Motasem Hamdan

**Affiliations:** 1 Faculty of Public Health, Al-Quds University, Jerusalem, P.O.Box 51000, occupied Palestinian territory

**Keywords:** Workplace violence, Hospitals, Health care workers, Nurses and physicians, Palestine

## Abstract

**Background:**

Violence against healthcare workers in Palestinian hospitals is common. However, this issue is under researched and little evidence exists. The aim of this study was to assess the incidence, magnitude, consequences and possible risk factors for workplace violence against nurses and physicians working in public Palestinian hospitals.

**Methods:**

A cross-sectional approach was employed. A self-administered questionnaire was used to collect data on different aspects of workplace violence against physicians and nurses in five public hospitals between June and July 2011. The questionnaires were distributed to a stratified proportional random sample of 271 physicians and nurses, of which 240 (88.7%) were adequately completed. Pearson’s chi-square analysis was used to test the differences in exposure to physical and non-physical violence according to respondents’ characteristics. Odds ratios and 95% confidence intervals were used to assess potential associations between exposure to violence (yes/no) and the respondents’ characteristics using logistic regression model.

**Results:**

The majority of respondents (80.4%) reported exposure to violence in the previous 12 months; 20.8% physical and 59.6% non-physical. No statistical difference in exposure to violence between physicians and nurses was observed. Males’ significantly experienced higher exposure to physical violence in comparison with females. Logistic regression analysis indicated that less experienced (OR: 8.03; 95% CI 3.91-16.47), and a lower level of education (OR: 3; 95% CI 1.29-6.67) among respondents meant they were more likely to be victims of workplace violence than their counterparts. The assailants were mostly the patients' relatives or visitors, followed by the patients themselves, and co-workers. Consequences of both physical and non-physical violence were considerable. Only half of victims received any type of treatment. Non-reporting of violence was a concern, main reasons were lack of incident reporting policy/procedure and management support, previous experience of no action taken, and fear of the consequences.

**Conclusions:**

Healthcare workers are at comparably high risk of violent incidents in Palestinian public hospitals. Decision makers need to be aware of the causes and potential consequences of such events. There is a need for intervention to protect health workers and provide safer hospital workplaces environment. The results can inform developing proper policy and safety measures.

## Background

Workplace violence in the health sector is a worldwide concern with healthcare workers being at high risk of being victims [1]. Violence includes any physical assault, verbal abuse or threatening behavior occurring in a workplace setting [2]. Both physical and non-physical violence against health care workers is a major problem affecting their health and productivity. Moreover, the consequences of workplace violence in the health sector have a significant impact on the effectiveness of health systems, especially in developing countries [3].

Although health care providers are increasingly concerned about the escalating incidence of workplace violence, there is a lack of evidence to support this concern due to low violence reporting rates [4]. In the Arab region also there is relatively limited research conducted on violence in health care settings [5-11].

Palestine is a country in chronic conflict and economic emergency [12]. The Palestinian Ministry of Health (MoH) is the main provider of health care services. The majority of the population is covered by the governmental health insurance scheme by which they are entitled to public services. This has increased the burden on limited public services, where most of public hospitals have high rates of service utilization and bed occupancy [13]. Besides routine care, these hospitals act as the main source of care for patients and injured people during political emergencies. Public hospitals are also known to suffer from many problems including understaffing and the frequent shortages of medicines and supplies which cause patients to wait for a long time before receiving services [14]. It is believed that these problems may cause violence against physicians and nurses. However, there remains a lack of adequate research evidence about the issue.

This study aims to assess the incidence, magnitude, and possible risk factors for workplace violence against nurses and physicians working in public hospitals in Palestine. It also examines the consequences of violence, professionals’ incident reporting patterns, and existing violence prevention and safety measures from health care workers’ perspectives. The study results can support the development of sound policy and strategies to prevent and manage work place violence against health workers in the country.

## Methods

### Study setting

There are eleven general MoH hospitals in the West Bank (WB). The study setting consisted of five of these public hospitals located in the five northern districts (Tulkarem, Nablus, Jenin, Qalqilya, Salfit) in the WB. The total number of the studied hospitals beds was 554 beds; about 41% of the total public beds in the WB [13].

### Study design

A cross-sectional design was adopted. Data were collected between June and July 2011. The study population (n = 928) consisted of all the licensed physicians (n = 292) and nurses (n = 636) who work on a full-time basis and with at least one year experience in these hospitals. Trainees or interns were excluded from the study. The population of the study was about 45% of the total physicians and nurses working in the MoH hospitals in the WB [13].

A proportionate stratified random sample was obtained from the study population. The total sample size was calculated from the study population (n = 928) based on the assumptions of α = 0.05, confidence interval 95%, and margin of error 0.05. The targeted 5 hospitals represented strata. The sample size from each stratum was proportional to its size in the study population, and within that the number of physicians and nurses were also proportionally calculated. This resulted in a sample of 271, composed of 84 physicians (31%) and 187 nurses (69%). The sample was randomly selected from each hospital.

### Study instrument

In this study work related violence is defined as any activity associated with the job or any event that occurs in the work environment that involves the intentional use of physical force or emotional abuse against an employee and results in physical or emotional injury and consequences [15,16]. Moreover, non-physical assault includes threat, sexual harassment, and verbal abuse [16].

The study instrument was prepared on the basis of the questionnaires used in two earlier studies [15,16]. The instrument was modified to fit the objectives of the study and the Palestinian hospitals context and was translated into Arabic. It was reviewed by five experts (nurses and physicians) to enhance its validity. Experts assessed the clarity, relevancy, comprehensiveness, and sensitivity of the tool to the culture. Basically, the expert comments were about the definitions of the violence, in specific the sexual violence, and clarity of some questions. The modified questionnaire was then pilot tested with 20 participants (physicians and nurses), who were excluded from the study sample.

The questionnaire gathered information on the following areas; socio-demographic data of the participants, exposure to physical and non-physical violence in the past 12 months, characteristics of perpetrators, magnitude and consequence of violence, incident reporting, availability of policies/procedures, training programmes, and safety measures in the workplace.

### Data collection

A self-administered questionnaire was distributed to 271 nurses and physicians. Permission to conduct the study and ethical approval were obtained from the MoH and Al-Quds University review board. Written consent was also obtained from participants after explaining the aim and assuring the confidentiality of the study; 249 questioners were completed, of which 240 were adequately completed. The general response rate was 88.7%; for physicians 95.2% and for nurses 85.5% respectively.

### Data analysis

Descriptive statistics were completed relating to the respondents’ characteristics. Pearson’s chi-square analysis was used to test the differences in exposure to violence (physical and non-physical violence) according to respondents’ characteristics. Crude odds ratios and 95% confidence intervals were used to assess potential associations between exposures to violence in general (yes/no) and respondents’ characteristics including gender, profession, age, years of experience, educational level, hospital department. Adjustment was then made for the same pre-mentioned covariates using a logistic regression model; the dependent variable being exposure to violence (yes, no). Data was analyzed using the Statistical Package for Social Sciences version 19. A *p* value <0.05 was considered statistically significant in the analysis.

## Results

The study respondents’ characteristics are provided in Table 1. The majority of respondents were females (59.2%), nurses (65.8%), younger than 41 years old (80.4%), holding a bachelor’s or higher degree (76.2%), with the experience more than five years (71.8%) in the profession, and work in shifts (68.3%). Moreover, most of them (78.7%) worked in medium or large sized (≥75beds) hospitals, the primary departments in which they regularly worked were medical/ surgical (21.7%), followed by emergency (20.4%), more than one department (11.7%), pediatrics (10.8%), and dialyses (10%).

**Table 1:** Characteristics of the respondents (n = 240)

**Characteristic**	**N**	**%**
**Gender**		
Male	98	40.8
Female	142	59.2
**Profession**		
Physician	82	34.2
Nurse	158	65.8
**Age groups**		
≤ 30 years	89	37.1
31-40 years	104	43.3
41-60 years	47	19.6
**Years of experience**		
1-5 years	68	28.3
6-10 years	87	36.3
11-15 years	45	18.8
Above 16 years	40	16.7
**Level of education**		
Diploma 2 years	57	23.8
Bachelor’s	163	67.9
Graduate studies	20	8.3
**Work in shifts**		
Yes	164	68.3
No	76	31.7
**Hospital size**		
Small, < 75 beds	51	21.3
Medium, 75–150 beds	109	45.4
Large, > 75 beds	80	33.3
**Department**		
Medical/Surgical	52	21.7
Emergency	49	20.4
Pediatrics	26	10.8
Split time more than one department	28	11.7
Dialyses	24	10.0
Intensive care	22	9.2
Operating/Recovery Room	14	5.8
Other department	25	10.4

### Incidence of workplace violence

In the 12 months prior to the survey, 80.4% of the respondents reported exposure to workplace violence. Of them, 20.8% reported exposure to physical violence, 59.6% reported non-physical violence that included 38.3% verbal abuse, 19.6% threats, and 1.7% sexual harassment. Meanwhile, 16.7% reported exposure to both physical and non-physical violence in the past 12 months (Table 2).

**Table 2:** Incidence of exposure to workplace violence (n = 240)

**Exposure to violence**	**Physical assault**		**Non-physical**								**Overall incidence**	
			**Threats**		**Verbal**		**Sexual**		**Total non-physical**			
	**N**	**%**	**N**	**%**	**N**	**%**	**N**	**%**	**N**	**%**	**N**	**%**
Yes	50	20.8	47	19.6	92	38.3	4	1.7	143	59.6	40	16.7
No	190	79.2	193	80.4	148	61.7	236	98.3	97	40.4	200	83.3

### Associations between exposure to violence and respondents’ characteristics

Table 3 shows the descriptive association between respondents’ characteristics and exposure to physical and nonphysical violence in the past 12 months. The results indicated that males (27.6%) had a significantly higher percentage of exposure to physical violence than females (P = 0.033), however, there was no significant difference in relation to reported non-physical violence by gender. Similarly, those whose education level was below bachelor’s degree reported a significantly higher percentage of physical violence incident (P = 0.022), but there was no significant difference in the percentage of non-physical violence in relation to the participants’ education level (P = 0.062). Respondents who had less than 10 years of experience reported a significantly higher percentage of both physical (P = 0.001) and non-physical (P < 0.001) violent incidents compared to respondents who had more years of experience. No significant differences were found in the percentages of reported physical or non-physical violent incidents and respondents’ age, profession, work in shifts, or hospital department (P > 0.05).

**Table 3:** Characteristics of exposures to physical and non-physical violence in the last 12 months (n = 240)

	**Physical violence**				Non-physical violence*			
	**N**	**%**	***χ***^***2***^	**P-value**	**N**	**%**	***χ***^***2***^	**P-value**
**Gender**								
Male	27	27.6	4.532	0.033	52	53.1	2.926	0.087
Female	23	16.2			91	64.1		
**Age**								
≤30 years	21	23.6	0.654	0.419	59	66.3	2.644	0.104
>30 years	29	19.2			84	55.6		
**Profession**								
Physician	19	23.2	0.413	0.521	51	62.2	0.353	0.553
Nurse	31	19.6			92	58.2		
**Education**								
< Bachelor's degree	18	31.6	5.234	0.022	40	70.2	3.483	0.062
≥ Bachelor's degree	32	17.5			103	56.3		
**Experience**								
≤10 years	42	27.1	10.410	0.001	115	74.2	38.793	P < 0.001
> 10 years	8	9.4			28	32.9		
**Work in shifts**								
Yes	37	22.6	0.937	0.333	96	58.5	0.236	0.627
No	13	17.1			47	61.8		
**Department**								
Inpatient	30	18.6	1.435	0.231	95	59.0	0.068	0.795
Outpatient	20	25.3			48	60.8		

* Nonphysical violence includes threats, verbal abuse and sexual harassment.

*χ*^*2*^*:* Pearson Chi-Square Test.

Table 4 shows the results of the unadjusted and multivariate-adjusted odds ratios for the exposure to violence (yes/ no) with their 95% confidence intervals according to the different characteristics of the respondents. Unadjusted crude odds ratios analysis indicates that exposure to violence incidents was significantly associated with respondents who were younger than 30 years old (P = 0.01), who had less than 10 years of experiences in the health sector (P < 0.001), and who had less than a bachelor’s degree of educational level (P = 0.022).

**Table 4:** Un-adjusted and multivariate-adjusted odds ratios for exposure to violence among respondents

	**Unadjusted**		**P-value**	Adjusted*		**P-value**
	**OR**	**95% CI**		**OR**	**95% CI**	
**Gender**						
Male	0.76	0.88-2.56	0.135	0.66	0.35-1.23	0.195
Female	1.0	Reference		1.0	Reference	
**Age**						
≤ 30 years	2.11	0.27-0.84	0.010	0.81	0.37-1.71	0.572
>30 years	1.0	Reference		1.0	Reference	
**Profession**						
Physicians	1.53	0.42-1.3	0.292	1.65	0.82-3.35	0.163
Nurses	1.0	Reference		1.0	Reference	
**Experience**						
< 10 years	7.05	0.08-0.26	0.001	8.03	3.91-16.47	0.001
≥ 10 years	1.0	Reference		1.0	Reference	
**Education**						
<Bachelor’s degree	2.3	0.22-0.86	0.016	2.94	1.29-6.67	0.010
≥Bachelor’s degree	1.0	Reference		1.0	Reference	
**Department**						
Inpatient	0.8	0.71-2.2	0.451	0.75	0.39-1.45	0.393
Outpatient	1.0	Reference		1.0	Reference	

* Adjusted for independent variables: Gender, profession, experience, education, age, and department.

OR: Odds ratios, CI: Confidence interval, Reference: reference category in the logistic regression model.

In comparison, the multivariate-adjusted odds ratios model shows that only two respondents’ characteristics remained significantly associated with exposure to work violence (Table 4). In particular, respondents who had less than 10 years of experience were 8 times more likely to be victims of violent incidents than those who had more experience (P < 0.001). Also, respondents who had less than bachelor’s degree level of education were almost 3 times more likely to be victims of violent incidents than those who had a higher educational level (P = 0.01).

### Characteristics of perpetrators

Table 5 shows the characteristics of perpetrators by type of violence. The respondents described perpetrators of physical violence as mainly males (76%), less than 36 years (88%), not impaired (58%) or impaired due to illness or prescribed medications (38%), perpetrators were mainly patient visitors/ relatives (48%), followed by patients/ clients (38%), and co-workers (14%) (including supervisors, physicians, nurses, others). In comparison, non-physical violence, perpetrators were mainly females (63.6%), of older age (36–60 years) (44.1%), not impaired (53.8%), and patients’ relatives (42%), coworkers (37.1%), and patients/clients (21%).

**Table 5:** Characteristics of perpetrators associated with physical and non-physical violence

	**Physical assailant**		Non-physical assailant*	
	**N**	**%**	**N**	**%**
**Gender**				
Male	38	76.0	52	36.4
Female	12	24.0	91	63.6
**Age group**				
≤18 years	18	36.0	31	21.7
19-35 years	26	52.0	49	34.3
36-60 years	6	12.0	63	44.1
**Impaired perpetrators**				
Yes, under the influence of illness or prescribed medicines	18	36.0	35	24.5
Yes, under influence of other drugs or alcohol	1	2.0	10	7.0
Not impaired	29	58.0	77	53.8
Not sure	2	4.0	21	14.7
**Relationship with perpetrators/ sources of violence**				
Patient/ clients	19	38.0	30	21.0
Visitors/ patient relatives	24	48.0	60	42.0
Co-workers (supervisors, physicians, nurses, others)	7	14.0	53	37.1

* Nonphysical violence includes threats, verbal abuse and sexual harassment.

### Magnitude and consequence of violent events

The results relating to the magnitude of the violent incidents showed that the majority of the participants reported exposure to a single violent physical (78%) and non-physical (71.3%) events; repeated events were respectively 22% and 28.7%. With regard to the timing of physical assaults, 48% of them happened in the evening, 20% in the night, 26% in the morning, and 6% were unsure about the time. Non-physical violence mainly (86%) happened face to face, 8.4% indicated through phone conversation and 5.6% by other means. Aggression mainly occurred in office stations (22%), patients’ rooms (14%), hallways (14%), and in the reception/ waiting area (10%).

The most frequent consequences of physical violence were anger (44%), depression (22%), fear or stress (14%), headache/ fatigue (8%), and frustration (6%). In non-physical violence consequences were anger (50.3%), headache/ fatigue (18.2%), depression (12.6%), and frustration (8.4%) (Table 6). Victims of physical assault were mainly treated by physicians (28%), psychiatrist (8%), or self treated (18%) and 46% received no treatment. Whereas for non-physical assault a large percentage had no treatment (62.9%), self treated (30%), or received psychiatric treatment (4.9%). While, 18% whom experienced physical violence reported persistent health problems as a result of the event, and 31.5% whom experienced non-physical violence reported persistent problems. Moreover, 48% of the victims of physical violence reported subsequent changes in their work status including restrictions in work (16%), work absences (16%) or transferred to another location (12%), and 59.2% of the victims of non-physical violence reported similar changes (Table 6).

**Table 6:** Characteristics and consequences of violent assault

	**Physical**		Non-physical*	
	**N**	**%**	**N**	**%**
**Symptoms/ feelings**				
Anger	22	44.0	72	50.3
Depression	11	22.0	18	12.6
Fear/stress	7	14.0	6	4.2
Headaches/ fatigue	4	8.0	26	18.2
Frustration	3	6.0	12	8.4
Irritability	2	4.0	3	2.1
Difficulty in sleeping	0	0.0	10	7.0
None	3	6.0	9	6.3
**Treatment by who**				
No treatment	23	46.0	90	62.9
Physician	14	28.0	3	2.1
I treat my self	9	18.0	43	30.1
Psychiatrist	4	8.0	7	4.9
**Persistent problems as a result**				
Yes	9	18.0	45	31.5
No	41	82.0	98	68.5
**Work changes as a result**				
No changes	26	52.0	85	59.4
Transfer to another location	6	12.0	7	2.9
Restrictions	8	16.0	26	10.8
Leave of absence	8	16.0	15	6.2
Other	2	4.0	10	4.2

* Nonphysical violence includes threats, verbal abuse and sexual harassment.

With regard to reporting violent events, 56.3% of the respondents did not report the incident, 20.4% of them orally reported to direct supervisors and 19.2% reported in writing. Of those who did not report the events, 32.5% indicated they did so because it was useless, from their experience no action would be taken, 20.8% indicated that it was not important, 20% were afraid of negative consequences or fear of feeling guilty or ashamed (14.6%), and lastly 10% did not know to whom they should report.

The majority of respondents (60%) indicated absence of procedures for reporting the violence, or any encouragement to report events (59%). Most of them also indicated that the hospitals had no specific policy/procedure or training programmes against workplace violence (85-95%). Only 13% indicated receiving training on any of the following issues: safety measures, dealing with violence, communication, or stress management. A very low percentage indicated availability of violence preventing policies/ procedures related to verbal abuse (18.3%), health and safety (16.6%), physical violence (12.5%), threat (12.5%), and sexual harassment (10.4%).

The respondents were also asked about the measures that exist in the work environment to deal with violence. The most frequently rated safety measures were the following: security personnel (75.4%), security alarms (36%), video monitoring systems (26.3%), cell phones (15%), and metal detectors (8.3%).

## Discussion

The main finding of the study was that 80.4% of the participants indicated exposure to workplace violence in the past 12 months. Despite some differences in the definition of violence, targeted professional groups, and methodology used, the study results are comparable with previous regional and international studies. In general, health workers in the Palestinian public hospitals have higher rate of exposure (80.4%) to both physical (20%) and non-physical violence (59.6%) than many other country studies [3,5,6,10,11,17]. The fact that the majority of respondents were exposed to some type of violence is also a matter of concern. Although this study did not investigate the perceived reasons for violence, we hypothesize that the high level of violence against health workers can be explained by the current state of public services including understaffing and inadequate working conditions, frequent shortages of medicines and supplies, overcrowded hospitals and delays in receiving care as well as unmet patient needs/expectations [13,14]. Furthermore, this situation is exacerbated, as the study results indicated, by lack of violence preventing strategies such as policy/procedures, training, and lack of adequate safety measures to protect health workers from violence in Palestinian public hospitals. Evidence from other studies showed that such conditions and factors can result in violence against health workers [5,17-19]. The dominant political instability and fragile economic conditions of the country could be other important causative factors. Difficult living conditions, frustration and stress in the daily life of Palestinians life probably increase the aggressive behavior against health workers; however, these factors were not examined in this study. AbuAlRub and colleagues [7] reported much higher level of violence against nurses in Iraq during political violence and economic instability. Increased violence against physicians in Israeli hospitals was also attributed to the deterioration of the economic and security situation [18].

In this study, physicians were slightly more exposed to violence than nurses, although, the difference was not statistically significant (P > 0.05). A study in Turkey [19] also showed the higher exposure of physicians to violence compared to other professions, but conversely in a study from Saudi Arabia nurses were significantly more exposed than physicians [9]. Culturally the medical profession in the Palestinian society is highly valued and dominant, which may account for this difference. Additionally, for that reason patients and relatives may hold higher expectations from physicians, dissatisfaction with health care can expose physicians to aggression more than other health care team members.

Certain characteristics have been found to increase the risk of workers being targets of workplace violence in the healthcare setting, including the workers’ gender, age, years of experience, marital status, and previous workplace violence training [20]. In this study, inconsistent with other studies in Lebanon, Egypt, and Saudi Arabia [6,10,11], no significant differences in the overall exposure to violence between males and females were found. However, males exposure to physical violence in general was significantly higher than females (P < 0.05). This was consistent with other studies from Arab countries in the region [5,10,11] and can be attributed to prevalent cultural norms rejecting disrespect to females in these societies. The logistic regression analysis, and in line with other studies [5,10,11,17] revealed that respondents with less experience in health sector and those with lower educational level were significantly associated with exposure to violence (P < 0.05). Other studies provided evidence that as the age of health workers increased, the frequency of violence committed against them decreased [5,10,11,17]. In this study, although significant association was found between respondents age groups and exposure to violence (P < 0.05), however, after adjustment by multiple logistic regression this association became insignificant (P > 0.05).

Detailed data on perpetrators were gathered in the study. Physical violence was mainly perpetrated by males of younger ages, and mainly those not impaired by illness, medications or substances. In comparison non-physical violence was mainly perpetrated by females of older age and mainly unimpaired persons. Available evidence show that men are more likely and physically capable of enacting physical violence than women who are more likely to enact verbal violence [11,16,20]. Also we can infer from our results that most of these incidents contained intentional violence and aggressive behaviors without perpetrators being under influence of disease or substances.

When we look at the magnitude of violence we can see that the majority were single events. Assaults were most likely (68%) to happen in the evening and night shifts (2 pm-8 am). Similar results were reported in studies in the region for examples Kuwait, Iraq, Saudi Arabia, and Egypt [5,7,9,10]. Higher rates of violence during this time can be also attributed to lower presence of hospital administration, and shortening of staff during the evening and night shifts that would require personnel to work alone [20]. Overloaded work demands place stress on human resources which would also increase conflict with patients and visitors. Consistence with research from Minnesota [16], most of the non-physical violence occurred face to face (86%) and in places where staff, patients, and relatives were in direct contact (60%) such as office stations, patients’ rooms, hallways, and reception/ waiting areas. This denotes inadequate communication skills between providers and recipients of care and weakness in dealing with violent acts or in how to engage the patients and families.

Many studies recognized emergency department as a particularly violent environment [21,22]. In the present study, not surprising, most of the events (28% of the physical and 24% of the non-physical assaults) happened in the emergency departments. These departments are usually attended by aggressive and stressed patients/ visitors and those patients who are impaired by substances who are more likely to commit violence against health workers [16,21,22].

Similar to many of the previous studies the patient relatives’ and patients were frequently reported as the main source of violence [3,7,9,11,16,17,19]. Nevertheless, a matter of concern was the proportion of violence created by colleagues or supervisors. About 14% of respondents who encountered physical violence and 37% encountered non-physical incidents from their co-workers specifically from physicians. This was found to be higher than some previous studies [6,11,16]. Understaffing, job stress, low job satisfaction are among possible factors that might lead to aggression towards colleagues and co-workers in Palestinian hospitals. Miedema and colleagues [23] found that victims of co-worker violence reported a loss of confidence in their clinical abilities and this subsequently influenced their mental well being [3]. In addition, the co-workers and colleagues violence has negative consequences on team cooperation and on the safety of patient care.

The magnitude of violence in Palestinian public hospitals represented in the frequency of exposure to more than one violent event during the past year (at least 22%) was higher than found in previous studies [5,16]. Moreover, 18% who experienced physical and 31.5% who experienced non-physical violence reported persistent problems as a result of the event. About half of those exposed did not receive any kind of treatment. This should be a matter of concern, especially as evidence shows that work related violence usually results in short and long term effects on the victims’ physical, psychological state, and professional performance [19,20]. Not surprisingly, most of the respondents indicated psychological and emotional feelings such as anger, fear, depression, stress and frustration. Other studies [24,25] showed that individuals who experience nonphysical violence, and endure feelings/symptoms over time, may be at risk for adverse mental health outcomes such as acute stress disorder or post-traumatic stress syndrome. In fact, about half of those who experienced physical or non-physical violence reported subsequent changes in their work status including restrictions in work, absence or subsequent transfer to another location. Apparently, this was much higher than the available evidence showed [9,16]. Attention should be given to violence deterrent policies and measures at the workplace as well as enforcement of the legal system after such events.

Low violence reporting level in this study (56.3%) was similar to previous studies [3,5,7,11,16,17,19,26]. The respondents attributed their reluctance to report due to lack of clear procedures for reporting and management encouragement to report. Respondents believed that reporting is useless because hospital management will not take any action besides, the fear of consequences such as blame or revenge of perpetrators. However, it is believed that socio-cultural norms and values of Palestinian society have a great impact. From experience it is known that in many cases incidents are not formally reported and disputes are settled through the tribal system rather than going to the court. Moreover, in many cases health workers consider this as part of the job, therefore tolerating the assailants, and do not feel that they should support reporting the events. The MoH should strengthen the incident reporting system in public hospitals and enforce laws to deter assaults against health workers as well as raising awareness in the community, and empower staff to cope with and report violence.

The study has some limitations, although a representative random sampling was used, due to time and resource restrictions, the study was limited to 5 public hospitals in the West Bank. Therefore these results may not be generalized to the whole hospital sector in Palestine. Moreover, the study used a retrospective self-reporting approach in data collection. This method depends on the ability of the participants to recall events in last 12 months previous to study, which might have potential biases.

## Conclusions

This study employed a comprehensive approach to identify the incidence, magnitude, consequences and potential risk factors for workplace violence against physicians and nurses in Palestinian public hospitals. The MoH needs to introduce policy and strategies for prevention and management of workplace violence, enhancement of incident reporting and follow up on reported events as well as providing adequate physical and psychological support to victims of health workplace violence. There is a need to encourage reporting and follow up on incidents as well as providing adequate physical and psychological support to victims of health workplace violence. The results of the study can serve the development of appropriate policy and strategies on workplace violence against health workers and also can serve as the basis for future studies in the country. Further research on workplace violence in other sectors, and on causes of violence in health care settings are needed.

## Abbreviations

MoH: Ministry of Health; WB: West Bank.

## Competing interest

The authors declare that they have no competing interests.

## Authors’ contributions

MK has made substantial contributions to the conception and design of the study, acquisition of data, analysis and interpretation of the data, and drafting the manuscript.

MH has supervised the study. He also has made substantial contributions to conception and design, analysis and interpretation of data, and drafting the manuscript. Both authors have given final approval of the version to be published.

## Pre-publication history

The pre-publication history for this paper can be accessed here:

http://www.biomedcentral.com/1472-6963/12/469/prepub
